# Characterization of Choroidal Layers in Normal Aging Eyes Using Enface Swept-Source Optical Coherence Tomography

**DOI:** 10.1371/journal.pone.0133080

**Published:** 2015-07-14

**Authors:** Mehreen Adhi, Daniela Ferrara, Robert F. Mullins, Caroline R. Baumal, Kathrin J. Mohler, Martin F. Kraus, Jonathan Liu, Emmerson Badaro, Tarek Alasil, Joachim Hornegger, James G. Fujimoto, Jay S. Duker, Nadia K. Waheed

**Affiliations:** 1 Department of Ophthalmology, New England Eye Center, Tufts Medical Center, Boston, Massachusetts, United States of America; 2 Department of Electrical Engineering and Computer Science and Research Laboratory of Electronics, Massachusetts Institute of Technology, Cambridge, Massachusetts, United States of America; 3 Department of Ophthalmology and Visual Sciences, University of Iowa, Iowa City, Iowa, United States of America; 4 Faculty of Physics, Ludwig-Maximilians-University Munich, Munich, Germany; 5 Pattern Recognition Lab and Graduate School in Advanced Optical Technologies, University Erlangen-Nuremburg, Erlangen-Nuremburg, Germany; Medical University of South Carolina, UNITED STATES

## Abstract

**Purpose:**

To characterize qualitative and quantitative features of the choroid in normal eyes using enface swept-source optical coherence tomography (SS-OCT).

**Methods:**

Fifty-two eyes of 26 consecutive normal subjects were prospectively recruited to obtain multiple three-dimensional 12x12mm volumetric scans using a long-wavelength high-speed SS-OCT prototype. A motion-correction algorithm merged multiple SS-OCT volumes to improve signal. Retinal pigment epithelium (RPE) was segmented as the reference and enface images were extracted at varying depths every 4.13μm intervals. Systematic analysis of the choroid at different depths was performed to qualitatively assess the morphology of the choroid and quantify the absolute thicknesses as well as the relative thicknesses of the choroidal vascular layers including the choroidal microvasculature (choriocapillaris, terminal arterioles and venules; CC) and choroidal vessels (CV) with respect to the subfoveal total choroidal thickness (TC). Subjects were divided into two age groups: younger (<40 years) and older (≥40 years).

**Results:**

Mean age of subjects was 41.92 (24-66) years. Enface images at the level of the RPE, CC, CV, and choroidal-scleral interface were used to assess specific qualitative features. In the younger age group, the mean absolute thicknesses were: TC 379.4μm (SD±75.7μm), CC 81.3μm (SD±21.2μm) and CV 298.1μm (SD±63.7μm). In the older group, the mean absolute thicknesses were: TC 305.0μm (SD±50.9μm), CC 56.4μm (SD±12.1μm) and CV 248.6μm (SD±49.7μm). In the younger group, the relative thicknesses of the individual choroidal layers were: CC 21.5% (SD±4.0%) and CV 78.4% (SD±4.0%). In the older group, the relative thicknesses were: CC 18.9% (SD±4.5%) and CV 81.1% (SD±4.5%). The absolute thicknesses were smaller in the older age group for all choroidal layers (TC p=0.006, CC p=0.0003, CV p=0.03) while the relative thickness was smaller only for the CC (p=0.04).

**Conclusions:**

Enface SS-OCT at 1050nm enables a precise qualitative and quantitative characterization of the individual choroidal layers in normal eyes. Only the CC is relatively thinner in the older eyes. *In-vivo* evaluation of the choroid at variable depths may be potentially valuable in understanding the natural history of age-related posterior segment disease.

## Introduction

Recent technological advances in ophthalmic imaging such as those in optical coherence tomography (OCT) reveal morphological changes of the choroid in addition to the retina in various pathological chorioretinal conditions. However, the fundamental aspects of the underlying pathophysiological mechanisms of these conditions remain unclear. It is still debatable whether the choroid is primarily involved, is affected either concurrently with the retina or is secondarily involved in these diseases [1,2]. Choroidal thinning has been associated with age-related macular degeneration (AMD) in both *in-vivo* and *post-mortem* studies [1,3–5]. Subfoveal total choroidal thickness measured on cross-sectional (B-scan) OCT has been found to significantly correlate with age and progressive subfoveal total choroidal thinning has been reported in normal aging eyes [6–9].

A detailed assessment of the choroid is still limited however to standard cross-sectional (B-scan) OCT imaging. Enface OCT imaging however allows high-definition, three-dimensional, depth-resolved choroidal reconstruction that documents details of the choroidal vasculature not readily appreciated on cross-sectional OCT imaging [10–13]. Although spectral-domain (SD)-OCT is standard in the clinical assessment and management of chorioretinal disorders, the assessment of the choroid may be compromised by the limited depth of penetration (~850nm), even when applying the enhanced depth imaging (EDI) method [14,15]. Swept-source (SS)-OCT is a modified Fourier-domain and depth resolved technology that offers potential advantages over SD-OCT including reduced sensitivity roll-off with imaging depth, higher detection efficiencies, improved imaging range, adaptability to longer imaging wavelengths of 1050nm that improve penetration of the choroid and higher image acquisition speeds [16]. SS-OCT employs a wavelength sweeping laser light source and a photodiode detector that records the interference of the backscattered light from the retina, while in SD-OCT a broadband light source is used and a spectrometer and line scan camera record the interference [17]. The improved performance of SS-OCT enables higher density raster scan protocols and deeper image penetration contributing to better visualization of choroidal detail on enface reconstructions [10–13].

The ability to better characterize the *in-vivo* choroidal morphology of normal eyes can lead to new insights related to the role of choroidal vasculature in both healthy and diseased states. This study describes three-dimensional qualitative and quantitative enface features of the individual choroid vascular layers in normal eyes using a novel, high-speed long-wavelength SS-OCT prototype.

## Methods

This study was performed under approved institutional review board protocols from the New England Eye Center (NEEC) and Massachusetts Institute of Technology. It adhered to the tenets of the Declaration of Helsinki and complied with the Health Insurance Portability and Accountability Act of 1996. Signed informed consent was obtained from all subjects before SS-OCT imaging. Healthy volunteers with no known history of any systemic condition or ophthalmic disease were examined at NEEC between October 2013 and April 2014. The subjects were medical students, trainees and staff at NEEC. All subjects received a complete ophthalmological examination including assessment of best-corrected visual acuity, biomicroscopy, intra-ocular pressure and a dilated fundus examination prior to imaging. Exclusion criteria were any known history of ophthalmological disease, refractive errors greater than 2 diopters, or any clinically visible changes on dilated fundus examination. Enrolled subjects were divided into two age groups: younger than 40 years of age (<40 years), or 40 years of age and older (≥40 years) for choroidal analysis.

A trained operator (MA) imaged all enrolled subjects using a SS-OCT prototype system operating at 1050nm. This device is similar to that described by Potsaid and colleagues [16,17]. It employs a commercially available 100kHz wavelength-swept semiconductor laser (Axsun Technologies Inc.) with a sweep bandwidth of ~100nm providing an axial resolution of ~6μm in tissue. The light incident on the eye is 1.9 mW, which is consistent with the American National Standard Institute (ANSI) standards for safe ocular exposure [18]. Three-dimensional 12x12 mm volumetric scans centered at the fovea with 400x400 axial scans were obtained, each acquired within 1.7 seconds. For each subject, at least two volumetric scans with orthogonal fast scan directions were acquired, then processed with the registration software for motion correction and merged into a single volumetric dataset to improve signal. An intensity and gradient based, semi-automatic algorithm was used to segment the retinal pigment epithelium (RPE)/Bruch’s membrane complex in order to generate a reference surface for enface display [19]. Enface SS-OCT scans of the RPE and choroid were extracted at varying depths every 4.13μm (1 pixel) from the RPE/Bruch’s membrane reference surface. Two retina specialists (DF, NKW) who are also both trained readers of the Boston Image Reading Center analyzed the qualitative and quantitative choroidal features (described below) in a masked fashion using enface SS-OCT images.

### Qualitative Assessment

Human donor eyes, obtained from the Iowa Lions Eye Bank following informed consent of the donors’ families, were fixed in 4% paraformaldehyde. A 4mm juxtamacular punch was taken from the posterior pole and embedded for cryostat sectioning as described previously [1]. Sections were labeled with vascular *Ulex europaeus* agglutinin-1 (UEA-I) and antibodies directed against collagen IV as described previously [20]. Briefly, sections were blocked in bovine serum albumin (1mg/mL) in 1x phosphate buffered saline with 1mM each of MgCl^2^ and CaCl^2^. After rinsing, sections were incubated with anti-collagen IV antibodies (Millipore, 1:200 dilution) and biotinylated UEA-I (1:100 dilution). After washing, antibodies and lectins were detected with Alexa-488 conjugated secondary antibodies and Texas red conjugated avidin. Sections were further counterstained with 4',6-diamidino-2-phenylindole, dihydrochloride (DAPI) and were imaged on a fluorescence microscope.

The individual choroidal vascular layers were characterized according to the vascular pattern documented on enface SS-OCT. Vessel density, diameter and orientation were taken into account. Both macular and extra-macular features were noted in the most representative enface scans of each individual layer. Individual choroidal layers were referred to as the choroidal microvasculature (including choriocapillaris, terminal arterioles and venules; CC) and the choroidal vessels (CV) including the inner choroid (medium-size choroidal vessels, or the so-called Sattler’s layer) and outer choroid (large-size choroidal vessels, or the so-called Haller’s layer). In addition, the RPE and the choroidal-scleral interface were also characterized.

The morphological characteristics of the labeled vasculature as seen on histology were used to help with qualitative assessment of the choroidal vasculature on the enface SS-OCT images of the subjects enrolled in the study.

### Quantitative Assessment

The thicknesses of CC and CV, as well as the subfoveal TC were measured by identifying the number of enface depth levels encompassed in each layer and multiplying the number of scans by 4.13 to convert the pixels into microns. These measurements will be referred herein as “absolute thicknesses”. The inner boundary of the choroid was defined as the RPE layer on enface SS-OCT image. A transition level between the CC and the CV was defined as the representative enface scan showing the features of both layers distributed in equal proportions in the macular area. The outer boundary of the choroid was defined as the choroidal scleral interface on enface SS-OCT image. Since there are no distinct morphological boundaries between the inner and outer choroid and this transition is rather a gradient of progressively larger vessels, the thicknesses of inner and outer choroid were not assessed independently in this study. The percentages that each individual choroidal layer (CC and CV) contributed to the TC were calculated. These measurements will be referred herein as “relative thicknesses”. The macular area was defined as a circular area of 6mm diameter centered at the fovea. The foveal area was defined as a circular area of 1mm diameter centered at the fovea [ETDRS Report 10 1991]. The total choroidal thickness was also measured on horizontal cross-sectional B-scans for method validation purposes (described below) using Image J (National Institutes of Health, Bethesda, MD; available at http://rsb.info.nih.gov/ij/index.html and accessed January 20, 2015).

The quantitative features (both absolute and relative thicknesses) were compared and their values were averaged between the two eyes of each subject. Following this, a comparison of the absolute and relative thicknesses was performed between the younger and older age groups.

### Method Validation

The quantitative assessment of the choroidal layers on SS-OCT underwent both internal and external validations to determine the reproducibility of the method of enface quantitative choroidal analysis described in this study. The inter-observer agreement when measuring the absolute thicknesses of CC, CV and TC was used for internal validation of the method. The agreement between the TC assessed on horizontal cross-sectional B-scans (current standard method [15]) and on enface imaging (study method) was used for external validation.

### Statistical Analysis

Intra-class correlation coefficients (ICC) and the methods of Bland and Altman were used for internal and external validations of the method. The reproducibility of absolute CC, CV and TC measurements was quantified using the bias and analyzed by the concordance coefficient with a 95% confidence interval. Unpaired student t-test was used to determine the difference in the averaged absolute thicknesses of CC, CV and TC and the relative thicknesses of CC and CV of both eyes in each subject between the younger and older age groups. Demographic statistics were presented as mean and standard deviation. All statistics were performed using Graph Pad Prism 5.0 software for Macintosh (GraphPad Software, La Jolla, CA) and SPSS Statistics (version 19, IBM Corp., Chicago, IL). A 95% confidence interval and a 5% level of significance were adopted and therefore, results with a p-value less than or equal to 0.05 were considered significant.

## Results

Fifty-two eyes of 26 consecutive healthy volunteers were enrolled in the study, all Caucasians, 8 males and 18 females, mean age 41.92 years (range 24 to 66 years). The younger group (<40 years) had 28 eyes (14 subjects), mean age 30.86 years (range 24 to 40 years). The older group (≥40 years) had 24 eyes (12 subjects), mean age 54.83 years (range 42 to 66 years). Enface SS-OCT images were generated from 12x12mm, three-dimensional volumetric SS-OCT datasets. Enface imaging allowed virtual sectioning of the datasets in consecutive scans 4.13 μm apart from each other, from the vitreous cavity to the sclera. Representative enface scans of the RPE, CC, CV, and choroidal-scleral interface were obtained.

### Qualitative Assessment

**Fig 1:**
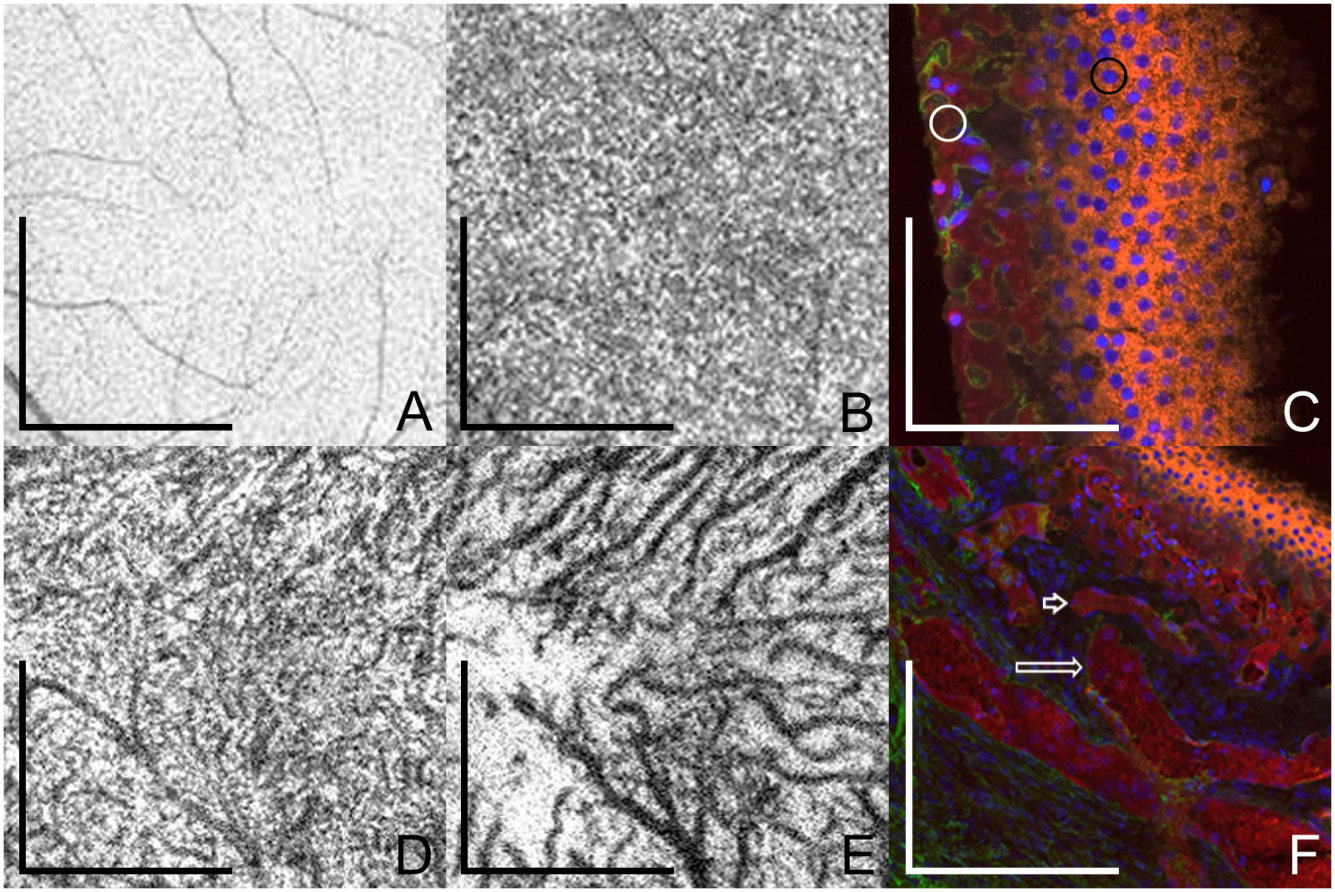
Enface swept-source optical coherence tomography (SS-OCT) describing the choroidal layers in a normal 40-year-old subject and post-mortem histological examination of the choroid in an 81-year-old subject. Enface SS-OCT scans were obtained at different levels. (**A**) Retinal pigment epithelium (RPE) appears as a homogeneous hyper-reflective layer; scale bar = 1.5 mm. The retinal vessels visible throughout scans are the shadows of the overlying retinal vessels (**B**) Choriocapillaris layer has a homogeneous reticular pattern, vessel lumens are indistinct on enface SS-OCT; scale bar = 1.5 mm. (**D**) Inner choroid has small caliber vessels in highly complicated but homogeneous disposition, with short vascular segments crossing over and under each other; scale bar = 1.5 mm. (**E**) Outer choroid has medium and large choroidal vessels, interwoven in the center of the macula and assuming clear radial distribution beyond the macular area towards the equator; scale bar = 1.5 mm. No distinct anatomic boundary can be determined between inner and outer choroid since vessel caliber and distribution change in a progressive gradient. (**C** and **F**) Histopathological section of the choroid from an 81-year-old female obtained with a 20x objective lens. Section is slightly oblique allowing more than one choroidal layer to be observed. Sections were labeled with UEA-I lectin (red) and the basal lamina marker anti-collagen IV (green). RPE nuclei are indicated by blue fluorescence from the 4',6-diamidino-2-phenylindole, dihydrochloride (DAPI) staining. (**C**) RPE (black circle) and choriocapillaris (white circle) are observed; scale bar = 50 μm. (**F**) Small caliber choroidal vessels (short arrow), and medium and large size choroidal vessels (long arrow) are observed deeper in the choroid; scale bar = 150 μm.

**Fig 2:**
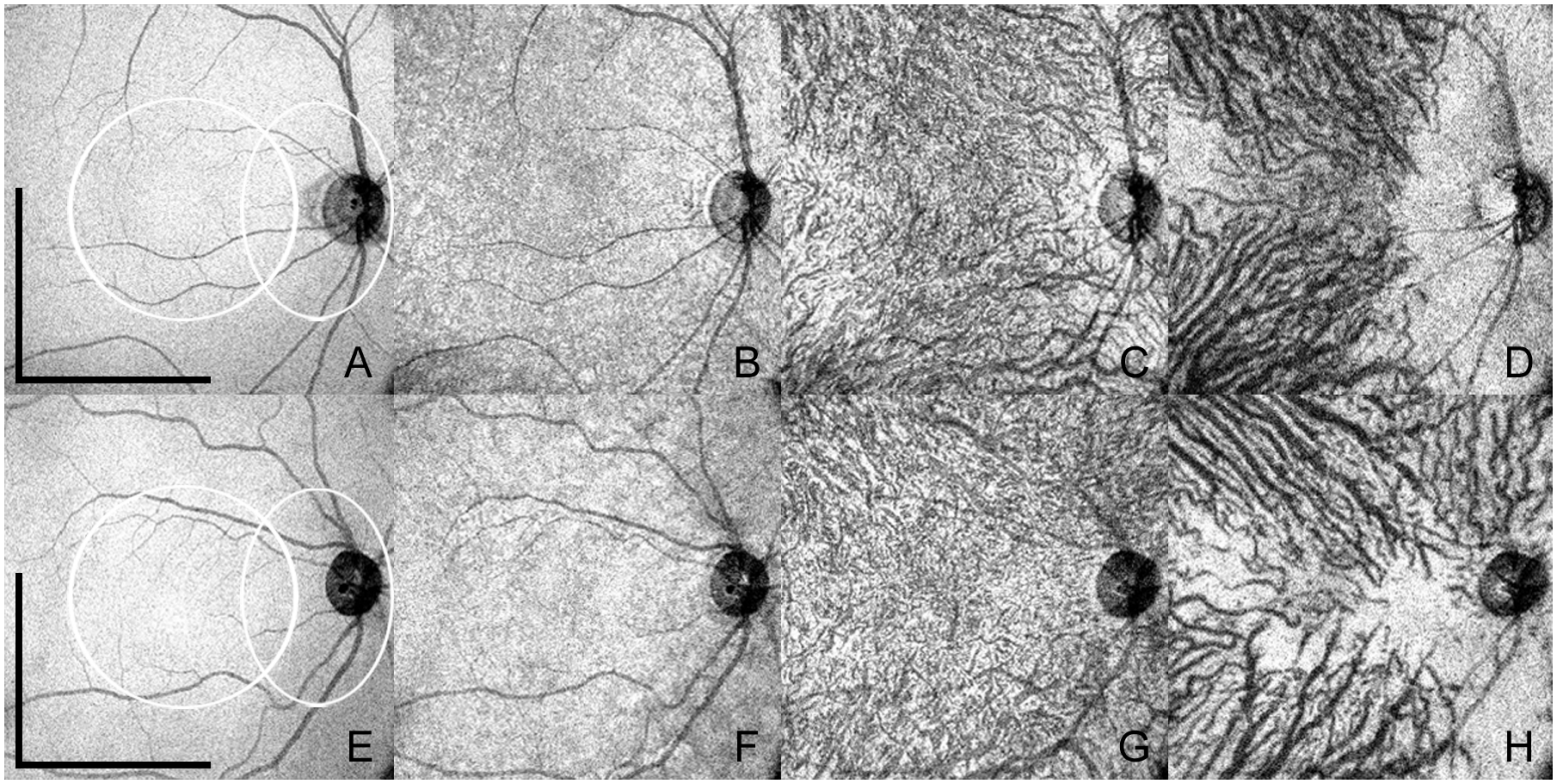
Enface swept-source optical coherence tomography (SS-OCT) of normal eyes of two different subjects belonging to the younger and older age groups. The upper row (**A**-**D**) shows enface SS-OCT of a 26 year old female where the total choroidal thickness measured 346μm. Scale bars (representing A-D) = 3mm. White circular area (representing A-D): macular area. White oval area (A-D): peripapillary area. The lower row (**E**-**H**) shows enface SS-OCT of a 47 year old female where the total choroidal thickness measured 201μm. Scale bars (representing E-H) = 3mm. White circular area (representing E-H): macular area. White oval area (E-H): peripapillary area. Retinal pigment epithelium (**A** and **E**) and individual choroidal layers, namely choriocapillaris (**B** and **F**), inner choroid (**C** and **G**) and outer choroid (**D** and **H**) have distinct features. Note variable choroidal thicknesses throughout the posterior pole, revealed by the visualization of the sclera in some areas, more commonly in the peripapillary region and temporal to the macula.

Enface SS-OCT image at the RPE level was characterized as a homogeneous, hyper-reflective monolayer throughout the scan including the macular and peripapillary areas. No RPE irregularities are present in enface scans of normal eyes. The only hypo-reflective elements corresponded to the optic disk and to the tomographic “shadowing” of the overlying normal retinal vessels (Figs 1A, 2A and 2E).

Enface SS-OCT images representative of the CC were characterized as a homogeneous reticular pattern throughout the scan including the macular and peripapillary areas and lying at a level external to the RPE and internal to the CV. Due to their reduced caliber and complex network, no distinct capillary vessels could be identified on the enface scans (Figs 1B, 2B and 2F). Microvascular choroidal anastomoses between the choriocapillaris and terminal arterioles or venules from the inner choroid are also present at this level. Since both choriocapillaris and microvascular anastomosis appear on enface SS-OCT as a complex reticular pattern, no tomographic distinction can be made between them.

Enface images at the level of the CV were also characterized. In the macular area, choroidal vessels appear as highly complicated, interwoven, short vascular segments crossing over each other. In the extra-macular area of enface scans, the choroidal vessels are straighter and fewer in number representing a radial distribution outward from the center of the macula towards the equator. Representative enface scans at the level of the inner choroid show thinner vessels, homogeneously distributed in the macular area through the topography of the retinal vascular arcades (Fig 2C and 2G). Representative enface scans at the level of the outer choroid show wider vessels, interwoven in the center of the macula but assuming clear radial distribution beyond the macular area towards the equator (Fig 2D and 2H). No distinct anatomic boundary can be determined between inner and outer choroid since vessel caliber and distribution change in a progressive gradient. Arteries and veins cannot be differentiated based on tomographic features. A peripapillary area of absent medium and large size choroidal vessels (but preserved choriocapillaris at a higher level) can be identified at the level of the outer choroid in all eyes. In some eyes this avascular area also extends inferiorly, which possibly corresponds to the angiographic description of the “watershed zone” [21,22] (Fig 2D and 2H).

The most representative enface SS-OCT scan of the choroidal-scleral interface was characterized by the absence of choroidal vessels in the foveal area. The sclera appears on enface scans as having a greyish, homogeneous surface. When evaluating progressively deeper levels in the volumetric, three-dimensional SS-OCT dataset, the scleral surface tends to appear in an irregular fashion suggesting variable thickness of the choroid throughout the posterior pole.

The morphological features of the different layers on enface SS-OCT and the representative histological specimens of additional donor eyes shared similarities on the fundamental characteristics that define each layer. Histologically, the choriocapillaris is a lobular network of smaller vessels, whereas the deeper choroid has progressively larger vessels that are ultimately supplied by or are drained by the short posterior ciliary arteries and vortex veins respectively. These patterns were observed consistently on the enface SS-OCT of the respective layers (Fig 1). Fig 2 represents the enface images at the level of RPE, CC, CV and the choroidal-scleral interface subjects belonging to the younger and an older age group.

### Quantitative Assessment

**Fig 3:**
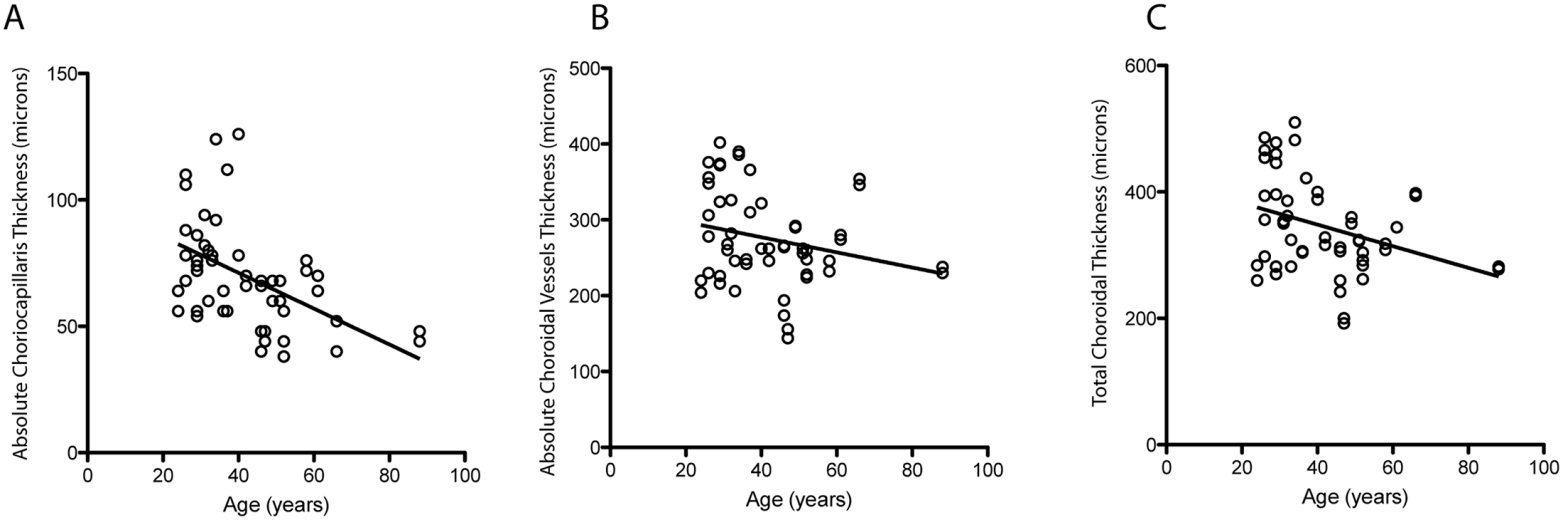
Scatter plots of absolute thicknesses in microns vs. age. (**A**) subfoveal total choroidal thicknesses, (**B**) absolute thickness of choriocapillaris **(C)** absolute thickness of choroidal vessels measured with enface swept source optical coherence tomography (SS-OCT).

**Fig 4:**
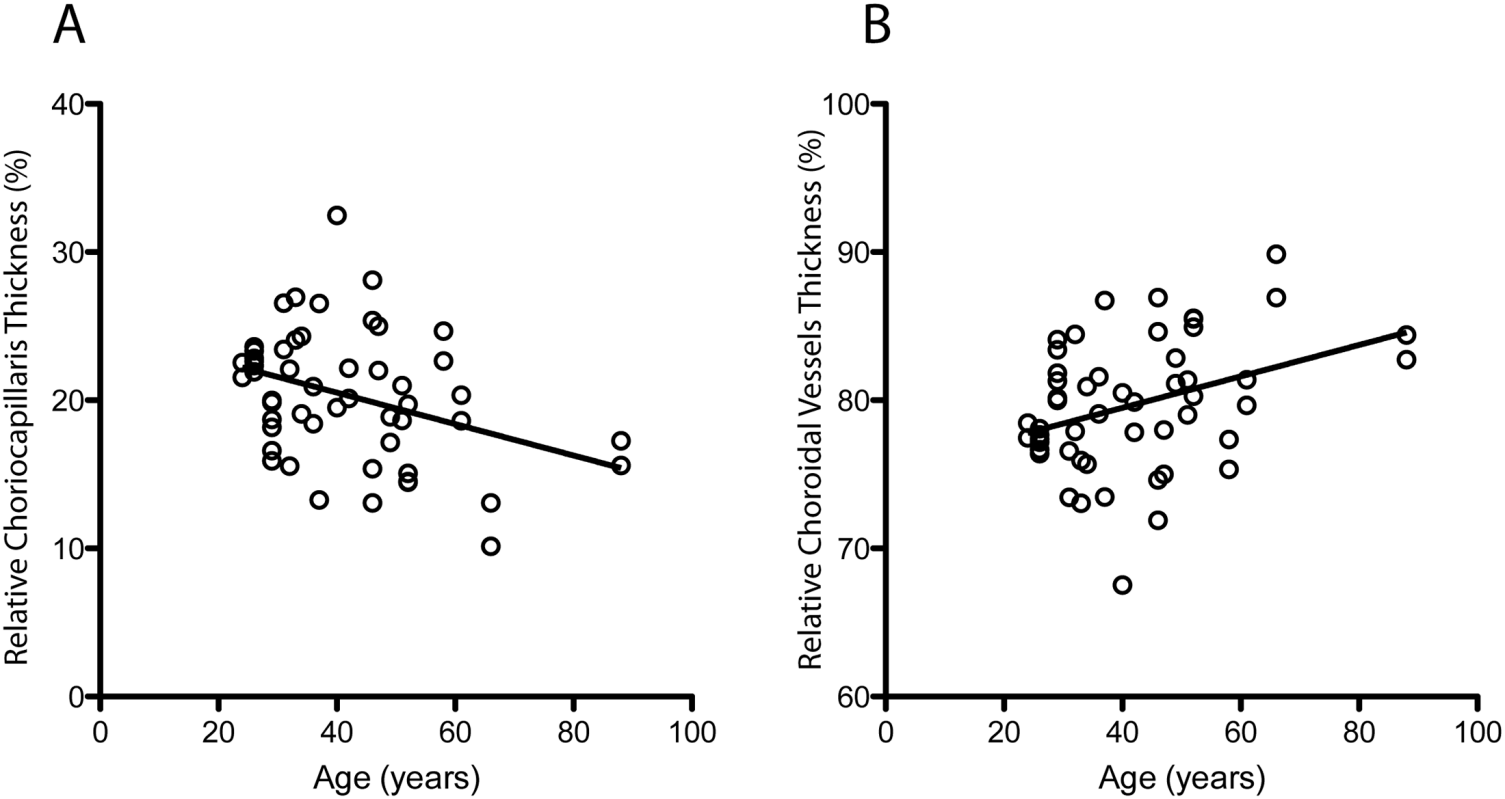
Scatter plots of relative thicknesses in percentages vs. age. **(A)** Relative thickness of choriocapillaris and **(B)** relative thickness of choroidal vessels measured with enface swept source optical coherence tomography (SS-OCT).

The TC, as well as the absolute and relative thicknesses of individual choroidal layers (CC and CV) were assessed on enface SS-OCT in each eye. Figs 3 and 4 demonstrate the scatter plots of the thickness values correlated with age. The absolute thicknesses of TC, CC and CV were well correlated between the left and right eyes (Table 1).

**Table 1:** Absolute and relative thicknesses of the choroid in the right and left eyes of the subjects. Mean values of subfoveal TC, as well as absolute and relative thicknesses of individual choroidal layers (CC and CV), measured with enface swept source optical coherence tomography (SS-OCT) including the intra-class correlation coefficient (ICC) for absolute thicknesses correlating the thickness values between the two eyes of each subject. n represents the number of eyes. Abbreviations: OD: right eye; OS: left eye; ICC: intra-class correlation coefficient for correlation between two eyes; SD: standard deviation; TC: subfoveal total choroidal thickness; CC: choriocapillaris; CV: choroidal vessels; N/A: non-applicable; CI: confidence interval.

	Mean OD (±SD) n = 26	Mean OS (±SD) n = 26	ICC (95% CI, p values)
TC Absolute Thickness (μm)	344.8 (±72.3)	345.3 (±78.8)	0.95 (0.93–0.97, <0.0001)
CC Absolute Thickness (μm)	68.0 (±18.0)	71.6 (±24.7)	0.97 (0.93–0.98, <0.0001)
CV Absolute Thickness (μm)	276.8 (±61.7)	273.7 (±64.0)	0.92 (0.90–0.95, <0.0001)
CC Percentage (%)	19.9 (±3.7)	20.7 (±5.0)	*N/A*
CV Percentage (%)	80.0 (±3.7)	79.3 (±5.0)	*N/A*

For further analysis, the TC and the absolute thicknesses of CC and CV between two eyes of each subject were averaged. Following this, the study cohort was divided between the younger and older age groups (Table 2). The means of the TC and the absolute and relative thicknesses of the individual choroidal layers (CC and CV) in the younger and older groups are depicted in Table 2. When comparing the absolute thicknesses between the younger and older groups, the mean values were significantly smaller in the older group for all layers (TC p = 0.006, CC p = 0.0003, CV p = 0.03). However, when comparing the relative thicknesses between the two age groups, only the CC was found to be significantly smaller in the older group (p = 0.04). On the contrary, the relative thickness of the CV was found to be significant higher in the older group (p = 0.04).

**Table 2:** Absolute and relative thicknesses of the choroid by age. Mean values of subfoveal TC, as well as absolute and relative thicknesses of individual choroidal layers (CC and CV) measured with enface swept source optical coherence tomography (SS-OCT) and compared using the unpaired student t-test in the younger (<40 years) and older (≥40 years) age groups. Average values of the two eyes from each subject were used for this comparison. n represents the number of values compared. Intra-class correlation coefficient (ICC) for inter-observer agreements of the measured absolute thicknesses is also shown. Abbreviations: ICC: intra-class correlation coefficient; SD: standard deviation; TC: subfoveal total choroidal thickness; CC: choriocapillaris; CV: choroidal vessels; N/A: non-applicable; CI: confidence interval.

	Mean Younger Group (±SD) n = 14	Mean Older Group (±SD) n = 12	ICC (95% CI, p values)	p values from unpaired student t-test
TC Absolute Thickness (μm)	379.4 (±72.0)	305.0 (±51.5)	0.94 (0.91–0.97, 0.001)	0.006
CC Absolute Thickness (μm)	81.2 (±17.0)	56.4 (±11.8)	0.91 (0.87–0.94, <0.0001)	0.0003
CV Absolute Thickness (μm)	298.1 (±60.0)	248.6 (±50.1)	0.92 (0.88–0.95, <0.0001)	0.03
CC Percentage (%)	21.5 (±2.8)	18.8 (±4.5)	N/A	0.04
CV Percentage (%)	78.4 (±2.8)	81.1 (±4.4)	N/A	0.04

### Method Validation

**Fig 5:**
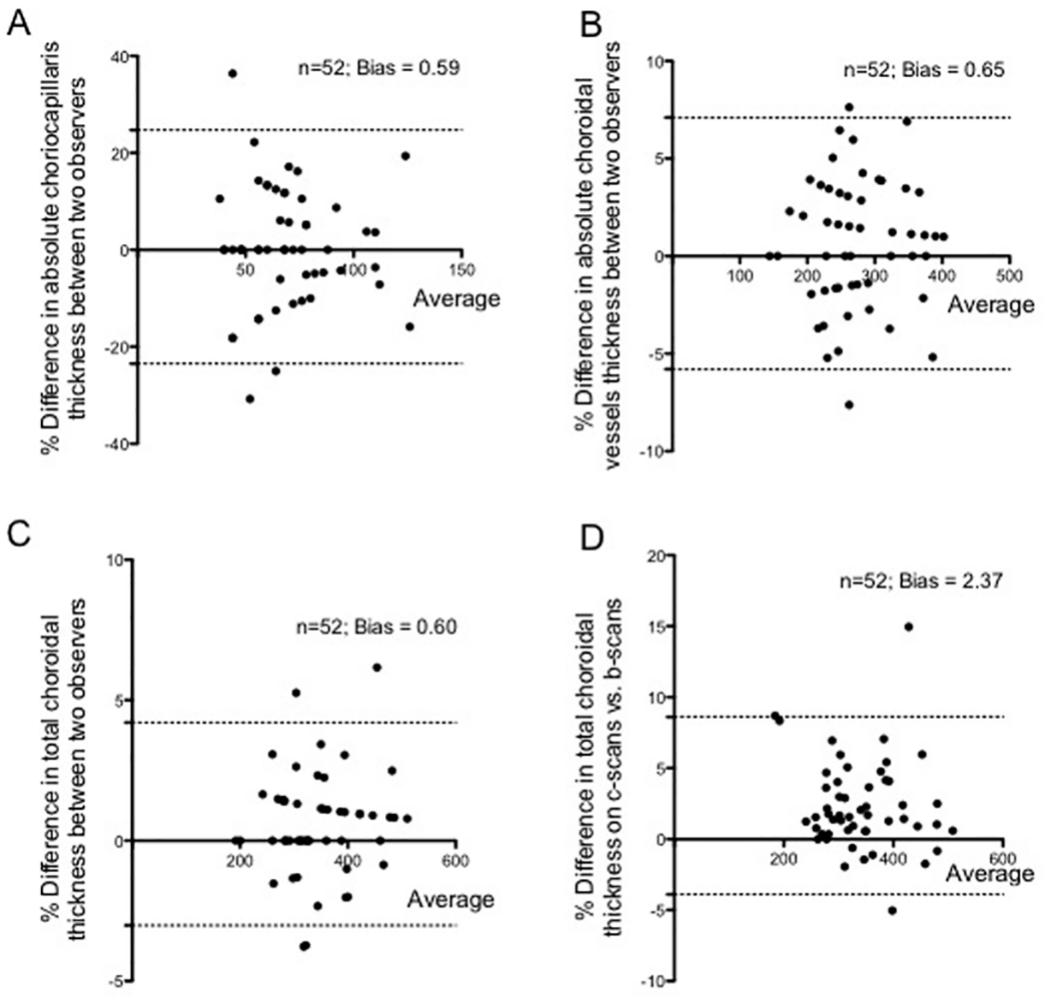
Bland Altman plots for internal and external validation of the method. **(A)** Graph showing an agreement between two independent observers for absolute thickness of choriocapillaris. **(B)** Graph showing an agreement between two independent observers for absolute thickness of choroidal vessels. **(C)** Graph showing an agreement between two independent observers for subfoveal total choroidal thickness. **(D)** Graph showing an agreement in subfoveal total choroidal thickness measured on cross-sectional swept source optical coherence tomography (SS-OCT) (B-scans) and enface SS-OCT images (C-scans). n represents the number of eyes and “bias” represents the results of the Bland Altman analysis. Note that **A-C** represent the internal validation of the method and **D** represents the external validation of the method.

The inter-observer agreement was considered for internal validation of the study method (Fig 5A–5C). The following ICC values were found for inter-observer reproducibility when measuring the TC and the absolute thicknesses of individual choroidal layers: TC = 0.94 (0.91–0.97, p = 0.001), CC = 0.91 (0.87–0.94, p<0.0001) and CV = 0.92 (0.88–0.95, p<0.0001) (Table 2).

The external validation of the method was assessed by the agreement between subfoveal TC measured on cross-sectional scans (B-scan) and that on enface SS-OCT images (C-scan) (Fig 5D). In the same trend, the ICC values were 0.90 (0.87–0.94, p = 0.001).

## Discussion

Histopathologic studies of the choroid have shown that subfoveal total choroidal thickness decreases with age, which has been repeatedly confirmed by studies using an *in-vivo* analysis of the choroidal thickness by cross-sectional SD-OCT [1,23,24]. While choroidal thinning on OCT has been associated with various pathological conditions such as AMD, age-related choroidal atrophy and high myopia, it is important to note that the total choroidal thickness also varies widely in the normal population at both the subfoveal and the extra-macular locations [7,24–27]. It may be challenging therefore, to establish a clear threshold between normal and pathologically thin choroids in a clinical setting. The results obtained in this study show in agreement with the literature a wide variability in subfoveal total choroidal thickness values in normal eyes belonging to both younger and older age groups. In addition, these results also revealed a large variability in the absolute thicknesses of individual choroidal layers in both age groups of the normal subjects.

Previous SS-OCT studies have not characterized in detail the *in-vivo* morphology of individual choroidal layers [11,12]. In the present study, enface SS-OCT permitted detailed, three-dimensional characterization of individual choroidal layers, from the RPE/Bruch’s membrane complex to the choroidal-scleral interface, demonstrating that an absolute and relative thickness of the choroidal microvasculature in normal eyes may also decrease with age, in addition to a decrease in the subfoveal total choroidal thickness. Future longitudinal studies following normal eyes over time are expected to further characterize changes in the qualitative and quantitative changes in the choroid with age. Normative values for *in-vivo* thickness of the choroidal microvasculature on SS-OCT have not been yet determined. Since enface SS-OCT cannot make a clear distinction between choriocapillaris, terminal arterioles and venules, the entire choroidal microvasculature (encompassing capillaries, feeder arterioles and draining venules) was assessed using the method employed in this study. Moreover, an alteration in the vascular blood flow may affect the measured thickness of the choroid. These considerations set the context for comparisons between *in-vivo* and *post-mortem* measurements of the choriocapillaris [1,21,22,28].

The current trend in the literature is to assess subfoveal total choroidal thicknesses with the same rationale applied for central retinal thicknesses, using absolute values. However, unlike choroidal thickness, total retinal thickness is markedly constant in the general population, and normative values have been determined using cross-sectional OCT [29–31]. The total choroidal thickness on the other hand show a variable distribution of thicknesses in *post-mortem* specimens, and the determination of normative values is challenged by its physiological variability [2,4]. The use of relative values has been established for a long time as the standard of care in the evaluation of the optic nerve rim with the cup-to-disk ratio, since absolute values of the optic disk diameter vary widely throughout populations [32,33]. Based on the presented findings, one may wonder whether a similar approach would be feasible for the clinical investigation of the choroidal and retinal thicknesses. When evaluating the relative thickness of each individual choroidal layer in normal eyes, the presented findings indicate that the choroidal microvasculature is significantly thinner in the older age group, while the choroidal vessels layer is relatively thicker. These results may propose an alternative strategy to assess the choroid as they suggest that relative choroidal thicknesses values may also have clinical relevance.

Interestingly, this study showed significant changes in the choroidal thicknesses between the two age groups using a cut-off age of 40 years. However, the authors only used the cut-off of 40 years of age and it is possible that since choroidal thinning is probably a gradual continuous process, it may begin at an even earlier age. The rationale of choosing the cut-off of 40 years was that other ophthalmological changes commonly related to an aging eye also have a gradual increase in incidence after 40 years of age, such as glaucoma and presbyopia. Although AMD typically becomes clinically evident in individuals older than 50 years, the results of the present study suggest that choroidal vascular changes might precede the clinical manifestation of the condition. The potential relevance of age-related relative thinning of the choroidal microvasculature as a risk factor for the development of AMD must be investigated in future studies involving both normal aging subjects and patients with early AMD.

Histopathologic studies of the choroid have shown that choroidal vessel density and diameter also decrease with age [23,28,34,35]. On enface SS-OCT, the vessel density could be subjectively observed by comparing the distribution of dark structures (OCT reconstruction of vessel lumen) and adjacent white areas (OCT reconstruction of dense structures and interstitial spaces) (Figs 1 and 2). The evaluation of choroidal vessel diameter was subjective and one given choroidal vessel could be compared only to adjacent choroidal vessels at the same depth as reported previously [13]. Nonetheless, this study reiterates the value of enface SS-OCT in evaluating different depths and the individual layers within the choroid (Fig 1).

The investigative enface SS-OCT method applied in this study was highly reproducible with high correlation between the observers as well as with the already established cross-sectional OCT (B-scan) method [15]. Hence, the authors believe that it is a valuable research method. In the clinical setting, however, its application is somewhat limited by the need for time consuming evaluation of enface scans by highly trained observers. The parameters proposed herein can be potentially applied to semi-automated or automated methods that could perform qualitative and quantitative characterization of the choroid in future [36].

In summary, enface SS-OCT imaging is a valuable tool for an *in-vivo* investigation of the individual choroidal layers, including the choroidal microvasculature. This imaging strategy may contribute to a better understanding of anatomical changes in the aging eye, and can potentially lead to new insights related to underlying physiopathological mechanisms of diseases such as AMD by indicating the exact level of primary age-related morphological changes within the choroid.
